# Associations between prenatal maternal exposure to per- and polyfluoroalkyl substances (PFAS) and polybrominated diphenyl ethers (PBDEs) and birth outcomes among pregnant women in San Francisco

**DOI:** 10.1186/s12940-020-00654-2

**Published:** 2020-09-16

**Authors:** Stephanie M. Eick, Elizabeth K. Hom Thepaksorn, Monika A. Izano, Lara J. Cushing, Yunzhu Wang, Sabrina Crispo Smith, Songmei Gao, June-Soo Park, Amy M. Padula, Erin DeMicco, Linda Valeri, Tracey J. Woodruff, Rachel Morello-Frosch

**Affiliations:** 1 Program on Reproductive Health and the Environment, Department of Obstetrics, Gynecology and Reproductive Sciences, University of California, San Francisco, CA USA; 2 Department of Environmental Health Sciences, Fielding School of Public Health, University of California, Los Angeles, CA USA; 3 Environmental Chemistry Laboratory, Department of Toxic Substances Control, California Environmental Protection Agency, Berkeley, CA USA; 4 Department of Biostatistics, Mailman School of Public Health, Columbia University, New York, NY USA; 5 Department of Environmental Science, Policy and Management and School of Public Health, University of California, Berkeley, CA USA

**Keywords:** Per- and poly-fluroalkyl substances, Polybrominated diphenyl ethers, Birth outcomes, Health disparities

## Abstract

**Background:**

Perfluoroalkyl substances (PFAS) and polybrominated diphenyl ethers (PBDEs) are used in consumer products for their water repellent and flame retardant properties, respectively. However, there is widespread prenatal exposure and concern about their potential harm to the developing fetus. Here, we utilized data from a demographically diverse cohort of women in San Francisco, CA to examine associations between prenatal exposure to PFAS and PBDEs with gestational age and birth weight for gestational age z-scores.

**Methods:**

Women included in this analysis were enrolled in the Chemicals in our Bodies (CIOB) cohort study (*N* = 506). PFAS and PBDEs were measured in serum obtained during the second trimester of pregnancy. Linear regression models were used to calculate crude and adjusted β coefficients for the association between PFAS and PBDE concentrations in tertiles and gestational age and birth weight z-scores. Individual PFAS and PBDE concentrations, as well as their sums, were examined in separate models.

**Results:**

The highest compared to lowest tertile of BDE-47 was associated with shorter gestational age (β = − 0.49, 95% confidence interval [CI] = − 0.95, − 0.02). Additionally, exposure to BDE-47 and BDE-99 in the middle tertile was also associated with a reduction in birth weight z-scores (β = − 0.26, 95% CI = -0.48, − 0.04; β = − 0.25, 95% CI = -0.47, − 0.04, respectively) compared to those in the lowest tertile of exposure. No consistent associations were observed between increasing PFAS concentrations and gestational age or birth weight z-scores.

**Discussion:**

Among a diverse group of pregnant women in the San Francisco Bay Area, we found non-linear associations between prenatal exposure to PBDEs during the second trimester of pregnancy and birth weight z-scores. However, most PFAS congeners were not associated with adverse birth outcomes. PFAS and PBDE concentrations were lower in our cohort relative to other studies. Future research should assess the effects of emerging and persistent PFAS and PBDEs on birth outcomes, as some congeners are being phased out and replaced by chemically similar structures.

## Background

Perfluoroalkyl substances (PFAS) and polybrominated diphenyl ethers (PBDEs) are persistent in the environment and ubiquitous due to their use in many consumer and industrial products. PFAS are in a wide range of products such as cookware, food containers, clothing, and fire-fighting foam due to their oil and water-repellent properties [1]. PBDE flame retardants have been applied to items such as home furniture, electronic devices and textiles [2]. Both PFAS and PBDEs can persist in the indoor environment [3], have long elimination half-lives in the human body [4, 5], can bioaccumulate in animals and humans, and become magnified in the food web [6].

In humans, studies have shown that PFAS and PBDEs travel through the placenta to the fetus [7–10]. PFAS and PBDEs have also been linked to adverse birth outcomes, including preterm birth (PTB) and low birth weight (LBW). For example, a recent systematic review of the effects of PFOA on fetal growth found that a 1 ng/mL increase in serum or plasma PFOA was associated with a − 18.9 g (95% confidence interval [CI] = − 29.8, − 7.9) decrease in birth weight [11]. Elevated levels of PFOA have also been associated with an increased risk of PTB [12]. In addition to PFOA, PFOS concentrations have been linked to a reduction in birth weight, gestational age and increased odds of PTB [12–14]. Despite the consistency for PFOA and PFOS, evidence for other PFAS has been mixed. Some studies suggest that higher concentrations of PFHxS [15] and PFNA [16] are associated with lower birth weight, while other studies have found null associations [14, 17]. The relationships between PFNA, gestational age, and PTB have also been inconsistent [13, 14].

Studies on the effects of PBDEs on birth outcomes are also mixed. In a recent meta-analysis, a one log unit increase in exposure to multiple PBDEs (BDE-47, BDE-99, BDE-100 and BDE-153) was associated with a − 50.6 g decrease in birth weight (95% CI = − 95.9, − 5.3) [18]. However, effect estimates are highly variable across individual studies [19–21]. Similar inconsistencies have been observed with gestational age. For example, two studies found no association between maternal serum concentrations of BDE-47 and BDE-99 and gestational age [20, 21], while another study found that elevated maternal serum concentrations of BDE-47 were associated with increased odds of PTB [22].

In response to health concerns, the US EPA worked with industry for a voluntary phase out of new production and import of PFOA in 2006 [23], PFOS by 3M in 2000–2002, and PentaBDE (which includes BDE-47 and BDE-99) [24]. California banned PBDEs in 2006, partially in response to studies showing that Californians had higher levels of PBDEs compared to other areas in the U.S. and globally [25–27]. This is likely due to the state’s strict flammability standard, which was revised in 2013 to improve fire safety without the use of flame retardant chemicals [28]. Since implementation of these regulatory reforms, blood serum concentrations of certain PBDEs and PFAS have subsequently declined [29–31]. However, while levels may have dropped, both PFAS and PBDEs remain ubiquitously present in the serum of pregnant women, and levels of certain chemicals, such as PFHxS [31] and PFNA [32], are not declining; a study of 65 pregnant women in the San Francisco Bay Area found that PFAS and PBDEs were detected in 98–100% and 56–90% of the maternal and cord blood samples, respectively [33]. A second study of pregnant women in California found that serum levels of BDE-47 and BDE-99 decreased from 2008 to 2011 and then plateaued from 2011 to 2014 [34].

To date, most studies have focused on specific PFAS and PBDE congeners, such as PFOA, PFOS, BDE-47, and BDE-99. However, there are continued widespread exposures to these persistent chemicals and newer chemicals are constantly being introduced to the market. Thus, there is uncertainty about the relationship between prenatal exposure to certain PFAS and PBDEs and adverse pregnancy outcomes. Therefore, the goal of this study was to characterize the association between prenatal exposure to PFAS and PBDEs and adverse birth outcomes. We included a number of PFAS and PBDE congeners not previously explored in this context, and hypothesized that higher maternal exposure to PFAS and PBDEs during pregnancy would be associated with a reduction in gestational age and birth weight z-scores.

## Methods

### Study population

Women included in the analysis were enrolled in the Chemicals in our Bodies (CIOB) study, a prospective birth cohort examining the effects of chemical and chronic psychosocial stress during pregnancy. All participants were recruited between 2014 and 2018 from prenatal clinics at the Zuckerberg San Francisco General Hospital (ZSFG) or Mission Bay (MB)/Moffitt Long (ML) hospitals during their 2nd trimester of pregnancy (between 12 and 28 weeks of gestation). Participants recruited from ZSFG were predominantly lower income women of color without private health insurance, whereas participants recruited from MB/ML were economically and racially/ethnically diverse and included women of higher socioeconomic status.

Women were eligible for inclusion in CIOB if they were older than 18 years of age, did not have a diagnosed pregnancy complication, were English or Spanish speaking, expecting a singleton birth, and planned to deliver at ZSFG or MB/ML. CIOB study protocols were approved by the Institutional Review Boards of the University of California, San Francisco (10–00861) and Berkeley (2010-05-04). All women provided written, informed consent prior to participating in the study. Further information describing recruitment methods for CIOB is available elsewhere [33].

### Covariates

Demographic information was collected via standardized questionnaire following informed consent and included: maternal age, maternal race/ethnicity (non-Hispanic [NH] White, NH Black, Hispanic, NH Asian), pre-pregnancy body mass index (BMI; < 18.5 kg/m^2^, 18.5–24.9 kg/m^2^, 25–29.9 kg/m^2^, ≥ 30 kg/m^2^), maternal education (<high school, high school completion or some college, college degree, >college degree or graduate education), parity (no prior births, one or more prior births), smoking status (never, ever, current), and country of birth (U.S., Other).

We additionally assessed food insecurity, as certain foods, food packaging, and reliance on food banks, have been associated with higher PFAS levels [35, 36]. Furthermore, food insecurity has been associated with adverse birth outcomes independent of pre-pregnancy BMI [37, 38]. This was done by asking participants five questions [39]. First, participants were asked how often within the last 12 months they (1) did not have enough money to purchase food or (2) could not afford to eat balanced and nutritious meals. Participants were additionally asked if, within the last 12 months, the following events ever occurred as a result of not having enough money to purchase food: (3) participants cut meals because there was not enough money for food, (4) participants ate less than they should, or (5) participants were hungry but chose not to eat. Women were considered food insecure if they reported yes to any of these events occurring.

### PFAS and PBDE exposure

Maternal whole blood was collected between 12 and 28 weeks gestation in red top tubes. Serum samples were centrifuged and aliquoted prior to storage at − 80 °C until analysis for 31 analytes (12 PFAS and 19 PBDEs). Analyses were performed by the Environmental Chemical Laboratory at the California Department of Toxic Substances Control (DTSC). Method detection limits (MDL) were calculated as 3 times the standard deviation of the blank concentrations for all PFAS and PBDEs [33]. Quality assurance plans were required for this study and were reviewed annually.

Serum preparation and extraction procedures were completed in the laboratory of DTSC. Briefly, serum was thawed, spiked with surrogate standards, denatured, and ran on an automated Biotage rapid trace SPE Work Station. Extractions were concentrated, cleaned up with acidic silica gel, reconcentrated, combined with internal standards, and analyzed for PBDEs by gas chromatography/high resolution mass spectrometry (GC-HRMS, DFS, Thermo-Scientific, Bremen, Germany) using isotope dilution [40]. PFAS were quantified by injection onto an automated on-line solid phase extraction method coupled to liquid chromatography and tandem mass spectrometry. Lipid content in maternal serum was also measured. Biospecimen collection instruments were chosen based on contamination testing results and instruments were tested for contamination prior to analyses. If contamination was found, other biospecimen collection and storage methods and instruments were used. Additional details regarding PFAS and PBDE measurement is provided elsewhere [33]. Values below the MDL were assigned the machine read value if a signal was detected. Chemicals that were below the MDL and no signal was obtained were coded as missing.

All wet-weight PBDE congeners were adjusted with lipid serum concentrations. For consistency with previous research, we included PFAS and PBDE congeners with ≥ 80% of samples above the MDL in our statistical analysis [41]. Based on this detection frequency cut-off, we examined associations between 5 PFAS (PFNA, PFOS, PFOA, Me-PFOSA-AcOH, and PFHxS) and 2 PBDEs (BDE-47 and BDE99) congeners in relation to birth outcomes. For these compounds, measurements below the MDL were assigned the machine-read value if a signal was detected. If a signal was not detected and the concentration was below the MDL, measurements were treated as missing.

### Birth outcomes

Participants’ medicals records were abstracted by trained study staff for the following measures: final gestational age at delivery in weeks, birth weight in grams, infant length, and infant head circumference. Gestational age on the medical record was calculated based on best-estimated date of delivery. If gestational age was missing on the medical record, it was imputed by subtracting date of last menstrual period from date of birth when this information was available (*N* = 6). Birth weight for gestational age z-scores were calculated using a U.S. population reference [42]. Birth weight z-scores are preferred over birth weight, as they account for gestational age at delivery and disentangle the effects of gestational age versus fetal growth.

### Statistical analysis

We computed descriptive statistics for all demographic characteristics, birth outcomes, and PFAS and PBDE concentrations within our study population. Spearman correlation coefficients were calculated to assess the relationships between log-transformed PFAS and PBDE concentrations.

We used linear regression models to calculate crude and adjusted β estimates and 95% CIs for the associations between tertiles of individual PFAS and PBDE congeners and gestational age and birth weight z-scores. We examined associations between tertiles of PFAS and PBDE concentrations and PTB using logistic regression. We also included the sum of 5 PFAS and 2 PBDE congeners (the PFAS and PBDEs with a minimum 80% detection frequency) as exposures in separate models. All demographic characteristics were explored as potential covariates in adjusted analyses. Covariates retained in final adjusted models were associated with birth weight or gestational age in bivariate analyses at *p* < 0.10 and had empirical evidence in the literature supporting an association with both the exposure and outcome. Final models were adjusted for maternal age, race/ethnicity, pre-pregnancy BMI, education, smoking status, parity, and food insecurity.

We conducted a number of sensitivity analyses to examine the robustness of our findings, and present the results in the supplemental information. First, we used linear regression to examine associations between tertiles of PFAS and PBDE concentrations with continuous birth weight restricted to term births only. We restricted our analysis to term births as birth weight is often confounded by gestational age. Second, we explored infant sex differences by examining the association between tertiles of individual PFAS and PBDE congeners and gestational age and birth weight z-scores stratified by infant sex, as prior research has shown sex-specific effects of endocrine disrupting chemicals. Lastly, we examined wet-weight PBDE concentrations with lipids as a covariate in relation to gestational age and birth weight z-scores.

All analyses were conducted in R Version 3.6.0. In all regression models, missing data for birth outcomes, covariates, and tertiles of PFAS and PBDE concentrations was handled using Multiple Imputation via Chained Equations (mice). Measurements that were below the MDL and did not have a machine read value (< 1% of measurements) were treated as missing. Using the mice approach, the variables with complete data are used to predict the missing values. We used the ‘mice’ package [43] to produce 10 datasets with complete information and these datasets were pooled in subsequent analyses.

## Results

There were 510 pregnant women enrolled in the CIOB study. Of this group, 4 participants withdrew prior to delivery, leaving a sample size of 506 for our analysis. The majority of women self-identified as Non-Hispanic (NH) White (38.6%) or Hispanic (34%), and over half of our analytic sample was between 25 and 34 years of age (Table 1). Approximately half of our study population had a normal pre-pregnancy BMI (47.6%) and one or more prior births (50%). Most women had a college degree (22.9%) or graduate education (37.5%). Distribution of demographic characteristics stratified by study hospital is provided in Table S1.

**Table 1:** Demographic characteristics of Chemicals in Our Body (CIOB) study population (*N* = 506)

	N (%)
Maternal Age	
Mean (SD)	32 (5.4)
Maternal Race/Ethnicity	
Non-Hispanic White	193 (38%)
Non-Hispanic Black	40 (8%)
Hispanic	173 (34%)
Asian/Pacific Islander	95 (19%)
*Missing*	5 (1%)
Pre-pregnancy Body Mass Index	
Underweight (< 18.5 kg/m^2^)	12 (2%)
Normal (18.5–24.9 kg/m^2^)	236 (47%)
Overweight (25–29.9 kg/m^2^)	128 (25%)
Obese (≥ 30 kg/m^2^)	89 (18%)
*Missing*	41 (8%)
Infant Sex	
Female	257 (51%)
*Missing*	27 (5.3%)
Parity	
One or More Previous Births	254 (50%)
*Missing*	7 (1%)
Maternal Education	
< High School	59 (12%)
High School Graduate or Some College	139 (27%)
College Degree	116 (23%)
Graduate Level Degree	185 (37%)
*Missing*	7 (1%)
Smoking Status	
Never	421 (83%)
Ever	60 (12%)
Current	10 (2%)
*Missing*	15 (3%)
Difficulty Paying for Basics	
Yes	146 (29%)
*Missing*	12 (2%)
Food Insecurity	
Yes	79 (16%)
Foreign Born	
Yes	209 (41%)
*Missing*	75 (15%)
Preterm Birth	
< 37 weeks gestation	41 (8%)
*Missing*	13 (3%)
	**Median (SD)**
Birth Weight (grams)	3300 (580)
*Missing*	18 (4%)
Gestational Age at Delivery (weeks)	39 (2.0)
*Missing*	13 (3%)

Note: percentages may not sum to 100 due to rounding

Abbreviations: *SD* standard deviation

The geometric mean of PFAS and PBDEs varied across compounds. The highest median observed was for BDE-47 and BDE-99 (median = 10.9 ng/gram lipid and 4.1 ng/gram lipid, respectively). Among the PFAS, PFOS had the highest median (1.93 ng/mL) followed by PFOA (0.76 ng/mL) (Table 2). BDE-99 and BDE-47 were highly correlated (Spearman’s R = 0.89; *p*-value < 0.01). PFAS concentrations were also moderately correlated with one another (Spearman’s R = 0.14 to 0.74; *p* value < 0.05 for each correlation) but not with PBDEs (Table 3).

**Table 2:** Distributions of PFAS (ng/mL) and PBDE (ng/g lipid) concentrations in maternal serum

		MDL (ng/mL)	% Above MDL	% Machine Readable	Median	IQR (25th Percentile, 75th Percentile)	95th Percentile	Maximum
**PFAS**								
Perfluorononanoic Acid	PFNA	0.06	98.8	99.6	0.30	(0.20, 0.44)	0.85	18.7
Perflucorooctane Sulfonic Acid	PFOS	0.07	100.0	100.0	1.93	(1.18, 3.13)	6.06	14.5
Perfluorooctanoic Acid	PFOA	0.13	99.8	100.0	0.76	(0.46, 1.12)	2.11	32.2
Methyl-perfluorooctane sulfonamide Acetic Acid	Me-PFOSA-AcOH	0.01	98.8	99.8	0.05	(0.03, 0.08)	0.19	1.79
Perfluorohexanesulphonic Acid	PFHxS	0.01	100.0	100.0	0.33	(0.20, 0.59)	1.52	4.94
*Total PFAS > 80% MDL*					3.68	(2.30, 5.72)	10.2	54.7
Perfluorodecanoic Acid	PFDeA	0.08	69.2	92.3	0.16	(0.12, 0.24)	0.50	3.87
Perfluoroundecanoic Acid	PFUdA	0.05	71.9	95.3	0.14	(0.09, 0.23)	0.40	1.26
Perfluorooctane Sulfonamide	PFOSA	0.02	2.37	42.5	<MDL	(<MDL, <MDL)	<MDL	0.11
Perfluorobutane Sulfonate	PFBS	0.03	0.79	52.0	<MDL	(<MDL, <MDL)	<MDL	0.10
Perfluoroheptanoic Acid	PFHpA	0.05	12.1	67.2	<MDL	(<MDL, <MDL)	0.17	0.49
Perfluorododecanoic Acid	PFDoA	0.20	2.17	57.9	<MDL	(<MDL, <MDL)	<MDL	0.48
Ethyl-perfluorooctane sulfonamido Acetic Acid	Et-PFOSA-AcOH	0.01	10.5	66.2	<MDL	(<MDL, <MDL)	0.05	0.09
**PBDE**								
2,2′,4,4′-tetra-bromodiphenyl ether	BDE-47	0.02	99.8	100.0	10.9	(7.32, 17.0)	39.7	364.3
2,2′,4,4′,5-penta-bromodiphenyl ether	BDE-99	0.02	84.4	99.0	4.1	(2.95, 5.91)	11.9	141.3
*Total PBDE > 80% MDL*					16.2	(11.9, 25.6)	54.2	505.6
2,2′,4′-tri-bromodiphenyl ether	BDE-17	0.02	0.00	14.4	<MDL	(<MDL, <MDL)	<MDL	<MDL
2,4,4′-tri-bromodiphenyl ether	BDE-28	0.02	5.34	64.0	<MDL	(<MDL, <MDL)	9.00	15.9
2,3′,4,4′-tetra-bromodiphenyl ether	BDE-66	0.02	0.20	9.88	<MDL	(<MDL, <MDL)	4.10	4.10
2,2′,3,4,4′-penta-bromodiphenyl ether	BDE-85	0.02	0.99	11.7	<MDL	(<MDL, <MDL)	<MDL	12.6
2,2′,4,4′,6-penta-bromodiphenyl ether	BDE-100	0.03	44.5	100.0	<MDL	(<MDL, 16.1)	16.1	90.2
2,2′,4,4′,5,5′-hexa-bromodiphenyl ether	BDE-153	0.03	57.9	95.1	9.59	(<MDL, 44.2)	44.2	232.1
2,2′,4,4′,5,6′-hexa-bromodiphenyl ether	BDE-154	0.03	0.79	14.0	<MDL	(<MDL, <MDL)	<MDL	15.9
2,2′,3,4,4′,5′,6-hepta-bromodiphenyl ether	BDE-183	0.03	0.20	20.2	<MDL	(<MDL, <MDL)	<MDL	4.17
2,2′,3,3′,4,4′,5,6′-octa-bromodiphenyl ether	BDE-196	0.03	0.00	15.8	<MDL	(<MDL, <MDL)	<MDL	<MDL
2,2′,3,3′,4,4′,6,6′-octa-bromodiphenyl ether	BDE-197	0.03	0.99	43.9	<MDL	(<MDL, <MDL)	<MDL	23.6
2,2′,3,3′,4,5′,6,6′-octa-bromodiphenyl ether	BDE-201	0.03	0.00	16.8	<MDL	(<MDL, <MDL)	<MDL	<MDL
2,2′,3,3′,5,5′,6,6′-octa-bromodiphenyl ether	BDE-202	0.03	0.00	12.7	<MDL	(<MDL, <MDL)	<MDL	<MDL
2,2′,3,4,4′,5,5′,6-octa-bromodiphenyl ether	BDE-203	0.03	0.00	13.0	<MDL	(<MDL, <MDL)	<MDL	<MDL
2,2′,3,3′,4,4′,5,5′,6-nona-bromodiphenyl ether	BDE-206	0.04	0.00	15.8	<MDL	(<MDL, <MDL)	<MDL	<MDL
2,2′,3,3′,4,4′,5,6,6′-nona-bromodiphenyl ether	BDE-207	0.04	0.79	53.6	<MDL	(<MDL, <MDL)	<MDL	13.1
2,2′,3,3′,4,5,5′,6,6′-nona-bromodiphenyl ether	BDE-208	0.04	0.00	27.5	<MDL	(<MDL, <MDL)	<MDL	<MDL
2,2′,3,3′,4,4′,5,5′,6,6′-deca-bromodiphenyl ether	BDE-209	0.14	2.77	58.1	<MDL	(<MDL, <MDL)	<MDL	75.5

Abbreviations: *PFAS* per- and polyfluoroalkyl substances, *PBDE* polybrominated diphenyl ethers, *MDL* method detection limit, *IQR* interquartile range

Note: % machine readable indicates the percent of PFAS and PBDE concentrations in which a signal was obtained. Median, IQR, 95th Percentile, and Maximum values were calculated with PFAS and PBDE concentrations below the MDL coded as missing

**Table 3:** Spearman correlation coefficients and corresponding *p*-values for natural log transformed PFAS (ng/mL) and lipid-adjusted PBDE (ng/g lipid) concentrations with > 80% detection in maternal serum (*N* = 506)

	PFNA	PFOS	PFOA	Me-PFOSA-AcOH	PFHxS	BDE-47	BDE-99
PFNA							
PFOS	0.71**						
PFOA	0.74**	0.60**					
Me-PFOSA-AcOH	0.16**	0.24**	0.14**				
PFHxS	0.56**	0.67**	0.69**	0.16**			
BDE-47	−0.06	−0.08*	−0.06	0.16**	−0.06		
BDE-99	−0.06	−0.08*	−0.06	0.10	−0.08*	0.89**	

*Indicates *p* < 0.10

**Indicates *p* < 0.05

In adjusted models, the highest compared to the lowest tertiles of PFOA, PFOS, PFNA, and total PFAS concentrations were moderately associated with a reduction in gestational age, although the confidence intervals included the null value (Table 4). The upper two tertiles of PFHxS concentrations were associated with an increase in gestational age relative to the lowest tertile (β = 0.42, 95% CI = 0.00, 0.85; β = 0.48, 95% CI = 0.03, 0.92, respectively) in unadjusted models (Table S2). These associations were attenuated in adjusted models (Table 4). Increasing concentrations of BDE-47, BDE-99, and total PBDE were associated with reduced gestational age in crude models (Table S2). After adjustment, associations between BDE-47 and gestational age persisted, where the highest compared to lowest tertile of BDE-47 was associated with a 0.49 week decrease in gestational age (95% CI = − 0.95, − 0.02) (Table 4).

**Table 4:** Adjusted linear regression coefficients and 95% confidence intervals for the associations between tertiles of PFAS (ng/mL) and PBDE (ng/g lipid) concentrations > 80% detection in maternal serum and gestational age in weeks and birth weight z-scores (*N* = 506)

		Gestational Age		Birth Weight Z Score	
	N	β	95% CI	β	95% CI
**PFAS**					
PFOA					
< 1.40	167	Reference		Reference	
1.40–0.96	172	−0.29	(−0.74, 0.17)	0.13	(−0.10, 0.35)
> 0.96	167	−0.10	(−0.63, 0.43)	0.12	(−0.14, 0.37)
PFOS					
< 1.40	167	Reference		Reference	
1.40–2.56	172	−0.19	(−0.64, 0.26)	− 0.01	(− 0.24, 0.22)
> 2.56	167	−0.08	(− 0.59, 0.43)	0.02	(− 0.23, 0.27)
PFHxS					
< 0.24	163	Reference		Reference	
0.24–0.49	176	0.28	(−0.18, 0.73)	0.08	(−0.15, 0.32)
> 0.49	167	0.09	(−0.47, 0.65)	0.15	(−0.12, 0.42)
PFNA					
< 0.24	171	Reference		Reference	
0.24–0.49	244	−0.28	(−0.71, 0.14)	0.01	(−0.20, 0.23)
> 0.49	91	−0.30	(−0.88, 0.28)	− 0.04	(− 0.32, 0.25)
Me-PFOSA-AcOH					
< 0.04	165	Reference		Reference	
0.04–0.06	171	0.12	(−0.32, 0.57)	−0.24	(− 0.46, − 0.02)
> 0.06	170	0.18	(−0.27, 0.63)	0.09	(−0.14, 0.32)
*Total PFAS*					
< 2.62	169	Reference		Reference	
2.62–4.83	171	−0.34	(−0.80, 0.12)	0.01	(−0.22, 0.24)
> 4.83	166	−0.21	(−0.72, 0.30)	0.06	(−0.19, 0.31)
**PBDE**					
BDE-47					
< 8.47	167	Reference		Reference	
8.47–14.22	172	−0.07	(−0.50, 0.35)	−0.26	(− 0.48, − 0.04)
> 14.22	167	− 0.49	(− 0.95, − 0.02)	−0.14	(− 0.37, 0.09)
BDE-99					
< 2.88	169	Reference		Reference	
2.88–4.71	170	0.07	(−0.35, 0.49)	−0.25	(− 0.47, − 0.04)
> 4.71	167	− 0.25	(− 0.71, 0.22)	−0.08	(− 0.31, 0.15)
*Total PBDE*					
< 11.38	171	Reference		Reference	
11.38–18.99	170	−0.13	(− 0.55, 0.29)	−0.26	(− 0.48, − 0.04)
> 18.99	165	− 0.41	(− 0.87, 0.05)	−0.09	(− 0.32, 0.14)

Abbreviations: *PFAS* per- and polyfluoroalkyl substances, *PBDE* polybrominated diphenyl ethers, *CI* confidence interval

Models adjusted for maternal age, maternal race/ethnicity, pre-pregnancy BMI, maternal education, smoking status, parity, and food insecurity

Me-PFOSA-AcOH was associated with a reduction in birth weight z-scores (β = − 0.24, 95% CI = -0.46, − 0.02 for the middle compared to lowest tertile). No associations were observed between other PFAS congeners (PFOA, PFOS, PFHxS, total PFAS) and birth weight z-scores (Table 4). Compared to those in the lowest tertile, individuals in the middle tertile of BDE-47, BDE-99, and total PBDE concentrations had reduced birth weight z-scores with a reduction of − 0.26 (95% CI = -0.48, − 0.04), − 0.25 (95% CI = -0.47, − 0.04), and − 0.26 (95% CI = -0.48, − 0.04), respectively (Table 4). Associations were similar in unadjusted models (Table S3).

Logistic regression models indicated that increasing tertiles of PFAS and PBDE concentrations were associated with a moderate, non-significant, increase in odds of PTB across all congeners examined (Table 5). The highest compared to lowest tertile of BDE-47 and total PBDE were associated with increased odds of PTB in unadjusted models (OR = 2.88, 95% CI = 1.27, 6.51; OR = 2.42, 95% CI = 1.09, 5.40, respectively) (Table S4). OR estimates were attenuated in adjusted models (Table 5).

**Table 5:** Adjusted logistic regression coefficients and 95% confidence intervals for the associations between tertiles of PFAS (ng/mL) and PBDE (ng/g lipid) concentrations > 80% detection in maternal serum and preterm birth (*N* = 506)

	N (PTB, FTB)	OR	95% CI
**PFAS**			
PFOA			
< 1.40	(11, 155)	Reference	
1.40–0.96	(14, 151)	1.79	(0.75, 4.28)
> 0.96	(16, 146)	2.37	(0.88, 6.38)
PFOS			
< 1.40	(13, 153)	Reference	
1.40–2.56	(13, 155)	1.21	(0.50, 2.91)
> 2.56	(15, 144)	1.87	(0.72, 4.88)
PFHxS			
< 0.24	(13, 148)	Reference	
0.24–0.49	(17, 157)	1.32	(0.58, 3.02)
> 0.49	(11, 147)	1.15	(0.38, 3.41)
PFNA			
< 0.24	(12, 159)	Reference	
0.24–0.49	(24, 220)	1.88	(0.83, 4.30)
> 0.49	(9, 82)	2.06	(0.72, 5.89)
Me-PFOSA-AcOH			
< 0.04	(13, 152)	Reference	
0.04–0.06	(15, 156)	0.96	(0.40, 2.28)
> 0.06	(18, 152)	1.14	(0.49, 2.63)
*Total PFAS*			
< 2.62	(12, 157)	Reference	
2.62–4.83	(17, 154)	1.70	(0.70, 4.17)
> 4.83	(16, 150)	2.08	(0.74, 5.82)
**PBDE**			
BDE-47			
< 8.47	(9, 157)	Reference	
8.47–14.22	(11, 158)	1.20	(0.47, 3.06)
> 14.22	(21, 137)	2.35	(0.97, 5.72)
BDE-99			
< 2.88	(13, 156)	Reference	
2.88–4.71	(11, 159)	0.78	(0.33, 1.88)
> 4.71	(21, 146)	1.25	(0.55, 2.84)
*Total PBDE*			
< 11.38	(10, 161)	Reference	
11.38–18.99	(13, 157)	1.21	(0.50, 2.95)
> 18.99	(22, 143)	1.88	(0.79, 4.48)

Abbreviations: *PFAS* per- and polyfluoroalkyl substances, *PBDE* polybrominated diphenyl ethers, *OR* odds ratio, *CI* confidence interval

Models adjusted for maternal age, maternal race/ethnicity, pre-pregnancy BMI, maternal education, smoking status, parity, and food insecurity

Results from models using wet-weight measurements of PBDEs, controlling for lipid levels, in relation to gestational age and birth weight z-scores were similar to models using lipid-adjusted PBDE measures (Table S5-S6). When term birth weight was the outcome of interest, no clear patterns emerged in relation to increasing PFAS concentrations. However, the middle compared to lowest tertile of BDE-47 was associated with a 100.85 gram reduction in term birth weight (95% CI = -200.56, − 1.11) after adjusting for confounders (Table S7). When stratifying by infant sex, the association between BDE-47 and a reduction in birth weight z-scores was stronger among females as compared to males (Table S8). No clear sex differences were observed when PFAS and PBDE congeners were modeled in relation to gestational age (Table S9).

## Discussion

In a demographically diverse cohort of pregnant women in San Francisco, California, we observed that women with elevated levels of BDE-47 and BDE-99 had shortened gestational age and a reduction in birth weight z-scores. We did not observe consistent associations between PFAS concentrations and gestational age or birth weight for gestational age z-scores. In our cohort, PFAS and PBDE concentrations were lower relative to other studies [12, 13, 34, 44].

The inverse association between gestational age and prenatal exposure to BDE-47 in the highest compared to lowest tertile is consistent with prior research. For example, a study in Texas found that women in the upper two quartiles of BDE-47 had increased odds of PTB [22]. In contrast, neither the CHAMACOS cohort in Salinas Valley nor a prospective birth cohort in China found associations between PBDE exposure and reduced gestational age [19, 21]. In our study, we observed no association between gestational age and BDE-99 or BDE-47 in the middle versus lowest tertile, which may suggest that levels of PBDE exposure higher than what we observed in our study are needed to observe gestational age effects. Additionally, our study found that exposure to BDE-47 and BDE-99 in the middle, but not the upper, tertile was associated with a reduction in birth weight z-scores, which suggests that the association between PBDEs and birth weight may not be linear. This supports previous findings which also reported non-linear associations between PBDE exposure and birth weight [21].

We observed no clear associations between PFAS, gestational age, and birth weight z-scores. Other studies have also observed inconsistent associations between exposure to certain PFAS congeners and continuous measures of gestational age or birth weight [14, 17, 45]. However, our analysis indicated that women in the upper two tertiles of PFOA, PFOS, PFHxS, and PFNA had moderately, albeit non-significant, increased odds of PTB relative to women in the lowest tertile. These findings align with those observed in the Project Viva cohort in Eastern Massachusetts, where women in the upper two quartiles of PFOS, PFNA, and PFHxS had slightly increased odds of PTB compared to the referent group [13]. Similarly, increasing exposure to PFOA and PFOS was associated with increased odds of PTB among 3535 women in Denmark [12].

Discrepancies across studies may be due to differences in the timing during pregnancy when PFAS is measured, as studies assessing temporal trends in PFAS levels during pregnancy have shown that levels tend to decrease with increasing gestational age [46]. Inconsistences between studies may also be due to temporal and geographic variability in PFAS levels, as well as phase-outs in the use, import and manufacturing of some PFAS in the U.S. and elsewhere [47]. Temporal exposure studies in the U.S. have shown that levels of some PFAS have been decreasing [31, 48]. Although use of several PFAS and PBDE compounds in our study have declined, many are persistent and bioaccumulative, and their presence in durable consumer products, such as furniture, building materials and flooring suggests that exposures are likely to last several decades [49]. In addition, PFAS and PBDE substitutes, such as GenX and PFBS, have not been well studied and are suspected to have similar harmful health effects as the chemicals they were designed to replace [50]. Future studies should characterize prenatal exposures and birth outcome effects of these substituted chemicals currently being used to achieve similar water and oil repellent properties and flame retardant features of phased out PFAS and PBDEs.

PFAS and PBDEs may be affecting fetal growth through several mechanisms. One hypothesis is that PFAS disrupt estrogen and thyroid hormones. Both estrogen and thyroid hormones are important for normal fetal growth and development, and maternal hypothyroidism has been associated with lower birth weight [51, 52]. Human in vitro studies have shown that PFAS, such as PFOS and PFOA, interfere with estrogen receptors [53–55]. Animal studies have also shown that PFAS disrupt normal thyroid function [56] and biosynthesis of thyroid and reproductive hormones [55, 57]. Another possible mechanism may be elevated oxidative stress, which has been associated with increased odds of PTB [58] and is hypothesized to be a downstream consequence of PFAS exposure [59]. PBDEs may affect birth outcomes through disruption of thyroid function. The thyroid hormone influences fetal growth and development throughout gestation. For example, during fetal development, the thyroid hormone stimulates intrauterine growth during the second half of pregnancy and mediates important changes in the fetus to help it mature and prepare for birth and delivery [60]. PBDEs have been shown to disrupt thyroid function [61, 62], particularly by inhibiting thyroid hormone deiodinase activity [63, 64]. Deiodinase activity in the placenta helps control levels of thyroid hormone in the fetus [65].

Our study has several important strengths. We measured several PFAS, including PFHxS, PFNA, Me-PFOSA-AcOH, that have not been as extensively studied as PFOA and PFOS. Our study population also included a racially/ethnically and socioeconomically diverse group of pregnant women. There is a need for more research among understudied minority populations that investigate how these groups may be disproportionately affected by environmental chemical exposures. Additionally, our study is larger than many previous studies examining the association between PBDEs and PFAS and birth outcomes. We also acknowledge several limitations of our study. Levels of some PBDEs and PFAS have been decreasing in the U.S. population over time and it is possible that the levels we measured in our study were at lower concentrations than the levels that would negatively impact birth outcomes. Our study may also have lacked adequate power to detect statistically significant effects. Furthermore, multiple tests were conducted and we cannot rule out the possibility of chance findings. Lastly, while we did adjust for many key covariates, residual confounding may still be a concern.

## Conclusions

Among a diverse group of pregnant women in the San Francisco Bay Area, we found non-linear associations between prenatal exposure to PBDEs during the second trimester of pregnancy, birth weight z-scores, and gestational age. With the exception of Me-PFOSA-AcOH, we did not observe an association between increasing PFAS exposure and gestational age or birth weight z-scores, although our results suggested that certain PFAS were slightly associated with PTB. Future studies should focus on emerging PFAS and PBDE congeners and substitutes, which are replacing those chemicals that are declining.

## Supplementary information

**Additional file 1: Table S1.** Demographic characteristics of Chemicals in Our Body (CIOB) study population stratified by delivery hospital. **Table S2.** Crude linear regression coefficients and 95% confidence intervals for the associations between tertiles of PFAS (ng/mL) and PBDE (ng/g lipid) concentrations in maternal serum and gestational age in weeks (*N* = 506). **Table S3.** Crude linear regression coefficients and 95% confidence intervals for the associations between tertiles of PFAS (ng/mL) and PBDE (ng/g lipid) concentrations in maternal serum and birth weight z-scores (*N* = 506). **Table S4.** Crude odds ratios and 95% confidence intervals for the associations between tertiles of PFAS (ng/mL) and PBDE (ng/g lipid) concentrations in maternal serum and preterm birth (*N* = 506). **Table S5.** Linear regression coefficients and 95% confidence intervals for the associations tertiles of wet weight PBDE (ng/mL) concentrations in maternal serum and gestational age in weeks (*N* = 506). **Table S6.** Linear regression coefficients and 95% confidence intervals for the associations tertiles of wet weight PBDE (ng/mL) concentrations in maternal serum and birth weight z-scores (*N* = 506). **Table S7.** Linear regression coefficients and 95% confidence intervals for the associations between tertiles of PFAS (ng/mL) and PBDE (ng/g lipid) concentrations in maternal serum and birth weight (grams) among full term births (*N* = 461). **Table S8.** Linear regression coefficients and 95% confidence intervals for the associations between tertiles of PFAS (ng/mL) and PBDE (ng/g lipid) concentrations in maternal serum and birth weight z-scores stratified by infant sex (*N* = 506). **Table S9.** Linear regression coefficients and 95% confidence intervals for the associations between tertiles of PFAS (ng/mL) and PBDE (ng/g lipid) concentrations in maternal serum and gestational age stratified by infant sex (*N* = 506).

## Data Availability

Per University of California, San Francisco Institutional Review Board approval, the data that support the findings of this study are restricted for transmission to those outside the primary investigative team. Data sharing with investigators outside the team requires IRB approval. Requests may be submitted to the Program on Reproductive Health and the Environment (PRHE).

## Abbreviations

PFAS: Per- and polyfluoroalkyl substances
PBDE: Polybrominated diphenyl ethers
CA: California
CIOB: Chemicals in Our Bodies
BDE-47: 2,2′,4,4′-tetra-bromodiphenyl ether
BDE-99: 2,2′,4,4′,5-penta-bromodiphenyl ether
CI: Confidence Interval
PTB: Preterm birth
LBW: Low birth weight
PFOA: Perfluorooctanoic Acid
PFOS: Perflucorooctane Sulfonic Acid
PFHxS: Perfluorohexanesulphonic Acid
PFNA: Perfluorononanoic Acid
BDE-100: 2,2′,4,4′,6-penta-bromodiphenyl ether
BDE-153: 2,2′,4,4′,5,5′-hexa-bromodiphenyl ether
U.S.: United States
EPA: Environmental Protection Agency
ZSFG: Zuckerberg San Francisco General
MB: Mission Bay
ML: Moffitt Long
NH: Non-Hispanic
BMI: Body mass index
MDL: Method detection limit
Me-PFOSA-AcOH: Methyl-perfluorooctane sulfonamide Acetic Acid
mice: Multiple imputation via changed equations
