# Supplementary material for: Associations between prenatal maternal exposure to per- and polyfluoroalkyl substances (PFAS) and polybrominated diphenyl ethers (PBDEs) and birth outcomes among pregnant women in San Francisco
Source: Environ Health. 2020 Sep 16;19:100. doi: 10.1186/s12940-020-00654-2 (PMC7495899; doi:10.1186/s12940-020-00654-2)
Supplement: Supplementary file 1 — Additional file 1: Table S1. Demographic characteristics of Chemicals in Our Body (CIOB) study population stratified by delivery hospital. Table S2. Crude linear regression coefficients and 95% confidence intervals for the associations between tertiles of PFAS (ng/mL) and PBDE (ng/g lipid) concentrations in maternal serum and gestational age in weeks (N = 506). Table S3. Crude linear regression coefficients and 95% confidence intervals for the associations between tertiles of PFAS (ng/mL) and PBDE (ng/g lipid) concentrations in maternal serum and birth weight z-scores (N = 506). Table S4. Crude odds ratios and 95% confidence intervals for the associations between tertiles of PFAS (ng/mL) and PBDE (ng/g lipid) concentrations in maternal serum and preterm birth (N = 506). Table S5. Linear regression coefficients and 95% confidence intervals for the associations tertiles of wet weight PBDE (ng/mL) concentrations in maternal serum and gestational age in weeks (N = 506). Table S6. Linear regression coefficients and 95% confidence intervals for the associations tertiles of wet weight PBDE (ng/mL) concentrations in maternal serum and birth weight z-scores (N = 506). Table S7. Linear regression coefficients and 95% confidence intervals for the associations between tertiles of PFAS (ng/mL) and PBDE (ng/g lipid) concentrations in maternal serum and birth weight (grams) among full term births (N = 461). Table S8. Linear regression coefficients and 95% confidence intervals for the associations between tertiles of PFAS (ng/mL) and PBDE (ng/g lipid) concentrations in maternal serum and birth weight z-scores stratified by infant sex (N = 506). Table S9. Linear regression coefficients and 95% confidence intervals for the associations between tertiles of PFAS (ng/mL) and PBDE (ng/g lipid) concentrations in maternal serum and gestational age stratified by infant sex (N = 506). [file 12940_2020_654_MOESM1_ESM.docx]

Table S1. Demographic characteristics of Chemicals in Our Body (CIOB) study population stratified by delivery hospital.

|  | **Zuckerberg San Francisco General Hospital (N=175)** | **Mission Bay/ Moffitt Long (N=305)** |
| --- | --- | --- |
|  | **N (%)** | **N (%)** |
| Maternal Age |  |  |
| Mean (SD) | 29 (6.0) | 34 (4.2) |
| Maternal Race/Ethnicity |  |  |
| Non-Hispanic White | 10 (6%) | 173 (57%) |
| Non-Hispanic Black | 15 (9%) | 20 (7%) |
| Hispanic | 129 (74%) | 37 (12%) |
| Asian/Pacific Islander | 17 (10%) | 74 (24%) |
| *Missing* | 4 (2%) | 1 (0%) |
| Pre-pregnancy Body Mass Index |  |  |
| Underweight (<18.5 kg/m^2^) | 3 (2%) | 7 (2%) |
| Normal (18.5-24.9 kg/m^2^) | 44 (25%) | 179 (59%) |
| Overweight (25-29.9 kg/m^2^) | 56 (32%) | 69 (23%) |
| Obese (> 30 kg/m^2^) | 60 (34%) | 26 (9%) |
| *Missing* | 12 (7%) | 24 (8%) |
| Infant Sex |  |  |
| Female | 92 (53%) | 162 (53%) |
| *Missing* | 9 (5%) | 0 (0%) |
| Parity |  |  |
| One or More Previous Births | 124 (71%) | 119 (39%) |
| *Missing* | 0 (0%) | 5 (2%) |
| Maternal Education |  |  |
| <High School | 53 (30%) | 6 (2%) |
| High School Graduate or Some College | 98 (56%) | 28 (9%) |
| College Degree | 17 (10%) | 94 (31%) |
| Graduate Level Degree | 3 (2%) | 174 (57%) |
| *Missing* | 4 (2%) | 3 (1%) |
| Smoking Status |  |  |
| Never | 144 (82%) | 259 (85%) |
| Ever | 15 (9%) | 41 (13%) |
| Current | 6 (3%) | 3 (1%) |
| *Missing* | 10 (6%) | 2 (1%) |
| Difficulty Paying for Basics |  |  |
| Yes | 107 (61%) | 32 (10%) |
| *Missing* | 10 (6%) | 2 (1%) |
| Food Insecurity |  |  |
| Yes | 59 (34%) | 16 (5%) |
| Foreign Born |  |  |
| Yes | 127 (73%) | 77 (25%) |
| *Missing* | 7 (4%) | 65 (21%) |
| Preterm Birth |  |  |
| <37 weeks gestation | 20 (11%) | 18 (6%) |
| *Missing* | 5 (3%) | 0 (0%) |
|  |  |  |
| Birth Weight (grams) | 3300 (660) | 3400 (510) |
| *Missing* | 5 (3%) | 0 (0%) |
| Gestational Age at Delivery (weeks) | 38 (2.1) | 39 (1.6) |
| *Missing* | 5 (3%) | 0 (0%) |

Note: percentages may not sum to 100 due to rounding.

Abbreviations: SD, standard deviation.

Table S2. Crude linear regression coefficients and 95% confidence intervals for the associations between tertiles of PFAS (ng/mL) and PBDE (ng/g lipid) concentrations in maternal serum and gestational age in weeks (N=506).

| **PFAS** | **N** | **β** | **95% CI** |
| --- | --- | --- | --- |
| PFOA |  |  |  |
| <1.40 | 167 | Reference | |
| 1.40-0.96 | 172 | -0.09 | (-0.53, 0.34) |
| >0.96 | 167 | 0.18 | (-0.25, 0.62) |
| PFOS |  |  |  |
| <1.40 | 167 | Reference | |
| 1.40-2.56 | 172 | 0.12 | (-0.31, 0.55) |
| >2.56 | 167 | 0.27 | (-0.18, 0.71) |
| PFHxS |  |  |  |
| <0.24 | 163 | Reference | |
| 0.24-0.49 | 176 | 0.42 | (0.00, 0.85) |
| >0.49 | 167 | 0.48 | (0.03, 0.92) |
| PFNA |  |  |  |
| <0.24 | 171 | Reference | |
| 0.24-0.49 | 244 | 0.01 | (-0.39, 0.42) |
| >0.49 | 91 | 0.03 | (-0.49, 0.56) |
| Me-PFOSA-AcOH |  |  |  |
| <0.04 | 165 | Reference | |
| 0.04-0.06 | 171 | 0.13 | (-0.31, 0.57) |
| >0.06 | 170 | 0.17 | (-0.26, 0.61) |
| *Total PFAS* |  |  |  |
| <2.62 | 169 | Reference | |
| 2.62-4.83 | 171 | -0.01 | (-0.45, 0.42) |
| >4.83 | 166 | 0.20 | (-0.24, 0.63) |
| **PBDE** |  |  |  |
| BDE-47 |  |  |  |
| <8.47 | 167 | Reference | |
| 8.47-14.22 | 172 | -0.18 | (-0.61, 0.24) |
| >14.22 | 167 | -0.80 | (-1.25, -0.36) |
| BDE-99 |  |  |  |
| <2.88 | 169 | Reference | |
| 2.88-4.71 | 170 | -0.02 | (-0.44, 0.41) |
| >4.71 | 167 | -0.59 | (-1.04, -0.14) |
| *Total PBDE* |  |  |  |
| <11.38 | 171 | Reference | |
| 11.38-18.99 | 170 | -0.23 | (-0.66, 0.20) |
| >18.99 | 165 | -0.72 | (-1.17, -0.28) |

Abbreviations: per- and polyfluoroalkyl substances, PFAS; polybrominated diphenyl ethers, PBDE; CI, confidence interval.

Table S3. Crude linear regression coefficients and 95% confidence intervals for the associations between tertiles of PFAS (ng/mL) and PBDE (ng/g lipid) concentrations in maternal serum and birth weight z-scores (N=506).

| **PFAS** | **N** | **β** | **95% CI** |
| --- | --- | --- | --- |
| PFOA |  |  |  |
| <1.40 | 167 | Reference | |
| 1.40-0.96 | 172 | 0.04 | (-0.18, 0.26) |
| >0.96 | 167 | -0.09 | (-0.32, 0.13) |
| PFOS |  |  |  |
| <1.40 | 167 | Reference | |
| 1.40-2.56 | 172 | -0.02 | (-0.25, 0.20) |
| >2.56 | 167 | -0.07 | (-0.29, 0.16) |
| PFHxS |  |  |  |
| <0.24 | 163 | Reference | |
| 0.24-0.49 | 176 | -0.05 | (-0.27, 0.18) |
| >0.49 | 167 | 0.01 | (-0.22, 0.23) |
| PFNA |  |  |  |
| <0.24 | 171 | Reference | |
| 0.24-0.49 | 244 | 0.00 | (-0.21, 0.21) |
| >0.49 | 91 | -0.17 | (-0.44, 0.10) |
| Me-PFOSA-AcOH |  |  |  |
| <0.04 | 165 | Reference | |
| 0.04-0.06 | 171 | -0.27 | (-0.50, -0.05) |
| >0.06 | 170 | 0.03 | (-0.19, 0.26) |
| *Total PFAS* |  |  |  |
| <2.62 | 169 | Reference | |
| 2.62-4.83 | 171 | -0.06 | (-0.28, 0.17) |
| >4.83 | 166 | -0.06 | (-0.29, 0.17) |
| **PBDE** |  |  |  |
| BDE-47 |  |  |  |
| <8.47 | 167 | Reference | |
| 8.47-14.22 | 172 | -0.26 | (-0.48, -0.03) |
| >14.22 | 167 | -0.22 | (-0.44, 0.01) |
| BDE-99 |  |  |  |
| <2.88 | 169 | Reference | |
| 2.88-4.71 | 170 | -0.25 | (-0.47, -0.02) |
| >4.71 | 167 | -0.16 | (-0.38, 0.07) |
| *Total PBDE* |  |  |  |
| <11.38 | 171 | Reference | |
| 11.38-18.99 | 170 | -0.27 | (-0.49, -0.05) |
| >18.99 | 165 | -0.18 | (-0.40, 0.05) |

Abbreviations: per- and polyfluoroalkyl substances, PFAS; polybrominated diphenyl ethers, PBDE; CI, confidence interval.

Table S4. Crude odds ratios and 95% confidence intervals for the associations between tertiles of PFAS (ng/mL) and PBDE (ng/g lipid) concentrations in maternal serum and preterm birth (N=506).

| **PFAS** | **N (PTB, FTB)** | **OR** | **95% CI** |
| --- | --- | --- | --- |
| PFOA |  |  |  |
| <1.40 | (11, 155) | Reference | |
| 1.40-0.96 | (14, 151) | 1.48 | (0.66, 3.31) |
| >0.96 | (16, 146) | 1.63 | (0.74, 3.59) |
| PFOS |  |  |  |
| <1.40 | (13, 153) | Reference | |
| 1.40-2.56 | (13, 155) | 1.07 | (0.48, 2.37) |
| >2.56 | (15, 144) | 1.34 | (0.62, 2.92) |
| PFHxS |  |  |  |
| <0.24 | (13, 148) | Reference | |
| 0.24-0.49 | (17, 157) | 1.25 | (0.59, 2.62) |
| >0.49 | (11, 147) | 0.95 | (0.42, 2.17) |
| PFNA |  |  |  |
| <0.24 | (12, 159) | Reference | |
| 0.24-0.49 | (24, 220) | 1.48 | (0.69, 3.16) |
| >0.49 | (9, 82) | 1.41 | (0.56, 3.56) |
| Me-PFOSA-AcOH |  |  |  |
| <0.04 | (13, 152) | Reference | |
| 0.04-0.06 | (15, 156) | 1.08 | (0.48, 2.44) |
| >0.06 | (18, 152) | 1.35 | (0.63, 2.93) |
| *Total PFAS* |  |  |  |
| <2.62 | (12, 157) | Reference | |
| 2.62-4.83 | (17, 154) | 1.42 | (0.65, 3.11) |
| >4.83 | (16, 150) | 1.34 | (0.60, 2.98) |
| **PBDE** |  |  |  |
| BDE-47 |  |  |  |
| <8.47 | (9, 157) | Reference | |
| 8.47-14.22 | (11, 158) | 1.30 | (0.51, 3.25) |
| >14.22 | (21, 137) | 2.88 | (1.27, 6.51) |
| BDE-99 |  |  |  |
| <2.88 | (13, 156) | Reference | |
| 2.88-4.71 | (11, 159) | 0.84 | (0.35, 1.98) |
| >4.71 | (21, 146) | 1.68 | (0.80, 3.55) |
| *Total PBDE* |  |  |  |
| <11.38 | (10, 161) | Reference | |
| 11.38-18.99 | (13, 157) | 1.31 | (0.54, 3.16) |
| >18.99 | (22, 143) | 2.42 | (1.09, 5.40) |

Abbreviations: per and poly-fluoroalkyl substances, PFAS; polybrominated diphenyl ethers, PBDEs; OR, odds ratio; CI, confidence interval; PTB, preterm birth; FTB, full term birth.

Models adjusted for maternal age, maternal race/ethnicity, pre-pregnancy BMI, maternal education, smoking status, parity, and food insecurity.

Table S5. Linear regression coefficients and 95% confidence intervals for the associations tertiles of wet weight PBDE (ng/mL) concentrations in maternal serum and gestational age in weeks (N=506).

|  |  | **Crude** | | **Adjusted** | |
| --- | --- | --- | --- | --- | --- |
| **PBDE** | **N** | **β** | **95% CI** | **β** | **95% CI** |
| BDE-47 |  |  |  |  |  |
| <8.47 | 167 | Reference | | Reference | |
| 8.47-14.22 | 172 | -0.26 | (-0.69, 0.16) | -0.17 | (-0.59, 0.26) |
| >14.22 | 167 | -0.83 | (-1.27, -0.38) | -0.51 | (-0.99, -0.03) |
| BDE-99 |  |  |  |  |  |
| <2.88 | 169 | Reference | | Reference | |
| 2.88-4.71 | 170 | -0.11 | (-0.55, 0.33) | -0.06 | (-0.49, 0.38) |
| >4.71 | 167 | -0.62 | (-1.09, -0.14) | -0.30 | (-0.79, 0.20) |
| *Total PBDE* |  |  |  |  |  |
| <11.38 | 171 | Reference | | Reference | |
| 11.38-18.99 | 170 | -0.22 | (-0.65, 0.21) | -0.11 | (-0.54, 0.31) |
| >18.99 | 165 | -0.72 | (-1.17, -0.27) | -0.38 | (-0.86, 0.10) |

Abbreviations: per-and polybrominated diphenyl ethers, PBDEs; OR, odds ratio; CI, confidence interval.

Crude models adjusted for lipid concentration. Adjusted models adjusted for lipid concentration, maternal age, maternal race/ethnicity, pre-pregnancy BMI, maternal education, smoking status, parity, and food insecurity.

Table S6. Linear regression coefficients and 95% confidence intervals for the associations tertiles of wet weight PBDE (ng/mL) concentrations in maternal serum and birth weight z-scores (N=506).

|  |  | **Crude** | | **Adjusted** | |
| --- | --- | --- | --- | --- | --- |
| **PBDE** | **N** | **β** | **95% CI** | **β** | **95% CI** |
| BDE-47 |  |  |  |  |  |
| <8.47 | 167 | Reference | | Reference | |
| 8.47-14.22 | 172 | -0.20 | (-0.42, 0.02) | -0.21 | (-0.42, 0.01) |
| >14.22 | 167 | -0.15 | (-0.37, 0.07) | -0.10 | (-0.34, 0.14) |
| BDE-99 |  |  |  |  |  |
| <2.88 | 169 | Reference | | Reference | |
| 2.88-4.71 | 170 | -0.30 | (-0.52, -0.07) | -0.32 | (-0.54, -0.10) |
| >4.71 | 167 | -0.12 | (-0.35, 0.11) | -0.06 | (-0.30, 0.17) |
| *Total PBDE* |  |  |  |  |  |
| <11.38 | 171 | Reference | | Reference | |
| 11.38-18.99 | 170 | -0.22 | (-0.45, 0.00) | -0.24 | (-0.46, -0.02) |
| >18.99 | 165 | -0.10 | (-0.33, 0.12) | -0.05 | (-0.29, 0.18) |

Abbreviations: per-and polybrominated diphenyl ethers, PBDEs; OR, odds ratio; CI, confidence interval.

Crude models adjusted for lipid concentration. Adjusted models adjusted for lipid concentration, maternal age, maternal race/ethnicity, pre-pregnancy BMI, maternal education, smoking status, parity, and food insecurity.

Table S7. Linear regression coefficients and 95% confidence intervals for the associations between tertiles of PFAS (ng/mL) and PBDE (ng/g lipid) concentrations in maternal serum and birth weight (grams) among full term births (N=461).

|  |  | **Crude** | | **Adjusted** | |
| --- | --- | --- | --- | --- | --- |
| **PFAS** | **N** | **β** | **95% CI** | **β** | **95% CI** |
| PFOA |  |  |  |  |  |
| <1.40 | 167 | Reference | | Reference | |
| 1.40-0.96 | 172 | 37.84 | (-65.57, 141.25) | 62.93 | (-42.94, 168.8) |
| >0.96 | 167 | 2.96 | (-101.46, 107.37) | 86.07 | (-36.31, 208.45) |
| PFOS |  |  |  |  |  |
| <1.40 | 167 | Reference | | Reference | |
| 1.40-2.56 | 172 | 24.80 | (-79.98, 128.58) | 1.62 | (-105.53, 108.77) |
| >2.56 | 167 | 1.02 | (-104.08, 106.12) | 14.26 | (-101.51, 130.03) |
| PFHxS |  |  |  |  |  |
| <0.24 | 163 | Reference | | Reference | |
| 0.24-0.49 | 176 | 45.69 | (-58.33, 149.72) | 82.2 | (-24.84, 189.23) |
| >0.49 | 167 | 44.30 | (-61.14, 129.74) | 75.71 | (-51.35, 202.78) |
| PFNA |  |  |  |  |  |
| <0.24 | 171 | Reference | | Reference | |
| 0.24-0.49 | 244 | 39.68 | (-55.17, 134.53) | 26.69 | (-73.74, 127.12) |
| >0.49 | 91 | -41.30 | (-166.39, 83.79) | -1.96 | (-138.13, 134.22) |
| Me-PFOSA-AcOH |  |  |  |  |  |
| <0.04 | 165 | Reference | | Reference | |
| 0.04-0.06 | 171 | -108.16 | (-211.40, -4.93) | -99.65 | (-202.26, 2.95) |
| >0.06 | 170 | 25.43 | (-78.83, 129.69) | 43.22 | (-62.86, 149.3) |
| *Total PFAS* |  |  |  |  |  |
| <2.62 | 169 | Reference | | Reference | |
| 2.62-4.83 | 171 | 14.27 | (-89.69, 118.23) | 8.55 | (-100.58, 117.68) |
| >4.83 | 166 | 0.34 | (-104.09, 104.76) | 33.51 | (-86.40, 153.41) |
| **PBDE** |  |  |  |  |  |
| BDE-47 |  |  |  |  |  |
| <8.47 | 167 | Reference | | Reference | |
| 8.47-14.22 | 172 | -104.74 | (-206.55, -2.92) | -100.84 | (-200.56, -1.11) |
| >14.22 | 167 | -103.20 | (-208.63, 2.23) | -54.53 | (-164.3, 55.24) |
| BDE-99 |  |  |  |  |  |
| <2.88 | 169 | Reference | | Reference | |
| 2.88-4.71 | 170 | -86.83 | (-189.31, 15.64) | -90.51 | (-190.84, 9.83) |
| >4.71 | 167 | -73.03 | (-177.73, 31.67) | -28.99 | (-137.49, 79.51) |
| *Total PBDE* |  |  |  |  |  |
| <11.38 | 171 | Reference | | Reference | |
| 11.38-18.99 | 170 | -109.95 | (-211.64, -8.25) | -104.41 | (-203.6, -5.22) |
| >18.99 | 165 | -84.07 | (-189.14, 21.00) | -33.53 | (-141.65, 74.59) |

Abbreviations: per and poly-fluoroalkyl substances, PFAS; polybrominated diphenyl ethers, PBDEs; CI, confidence interval.

Note: bold indicates p<0.05.

Models adjusted for maternal age, maternal race/ethnicity, pre-pregnancy BMI, maternal education, smoking status, parity, and food insecurity.

Table S8. Linear regression coefficients and 95% confidence intervals for the associations between tertiles of PFAS (ng/mL) and PBDE (ng/g lipid) concentrations in maternal serum and birth weight z-scores stratified by infant sex (N=506).

|  |  | **Male (N=233)** | |  | **Female (N=273)** | |
| --- | --- | --- | --- | --- | --- | --- |
| **PFAS** | **N** | **β** | **95% CI** | **N** | **β** | **95% CI** |
| PFOA |  |  |  |  |  |  |
| <1.40 | 69 | Reference | | 90 | Reference | |
| 1.40-0.96 | 79 | 0.07 | (-0.25, 0.39) | 82 | 0.18 | (-0.14, 0.51) |
| >0.96 | 74 | 0.12 | (-0.26, 0.49) | 85 | 0.15 | (-0.23, 0.53) |
| PFOS |  |  |  |  |  |  |
| <1.40 | 77 | Reference | | 84 | Reference | |
| 1.40-2.56 | 71 | -0.11 | (-0.43, 0.22) | 90 | 0.08 | (-0.25, 0.42) |
| >2.56 | 74 | 0.07 | (-0.28, 0.42) | 83 | -0.03 | (-0.40, 0.34) |
| PFHxS |  |  |  |  |  |  |
| <0.24 | 67 | Reference | | 90 | Reference | |
| 0.24-0.49 | 85 | 0.14 | (-0.18, 0.47) | 82 | 0.06 | (-0.28, 0.41) |
| >0.49 | 50 | 0.14 | (-0.25, 0.52) | 85 | 0.22 | (-0.18, 0.63) |
| PFNA |  |  |  |  |  |  |
| <0.24 | 70 | Reference | | 90 | Reference | |
| 0.24-0.49 | 112 | 0.02 | (-0.28, 0.33) | 119 | 0.01 | (-0.30, 0.32) |
| >0.49 | 40 | 0.01 | (-0.42, 0.43) | 48 | -0.08 | (-0.50, 0.33) |
| Me-PFOSA-AcOH |  |  |  |  |  |  |
| <0.04 | 78 | Reference | | 84 | Reference | |
| 0.04-0.06 | 65 | -0.18 | (-0.50, 0.14) | 96 | -0.27 | (-0.58, 0.04) |
| >0.06 | 79 | -0.02 | (-0.34, 0.30) | 77 | 0.20 | (-0.13, 0.53) |
| *Total PFAS* |  |  |  |  |  |  |
| <2.62 | 74 | Reference | | 86 | Reference | |
| 2.62-4.83 | 75 | 0.04 | (-0.29, 0.37) | 85 | 0.01 | (-0.33, 0.35) |
| >4.83 | 72 | 0.17 | (-0.20, 0.54) | 86 | -0.01 | (-0.39, 0.36) |
| **PBDE** |  |  |  |  |  |  |
| BDE-47 |  |  |  |  |  |  |
| <8.47 | 74 | Reference | | 91 | Reference | |
| 8.47-14.22 | 75 | -0.10 | (-0.41, 0.21) | 85 | -0.38 | (-0.69, -0.07) |
| >14.22 | 71 | -0.06 | (-0.39, 0.27) | 81 | -0.23 | (-0.57, 0.10) |
| BDE-99 |  |  |  |  |  |  |
| <2.88 | 68 | Reference | | 93 | Reference | |
| 2.88-4.71 | 82 | -0.22 | (-0.53, 0.09) | 81 | -0.26 | (-0.58, 0.05) |
| >4.71 | 72 | 0.02 | (-0.32, 0.37) | 83 | -0.21 | (-0.54, 0.12) |
| *Total PBDE* |  |  |  |  |  |  |
| <11.38 | 71 | Reference | | 92 | Reference | |
| 11.38-18.99 | 76 | -0.07 | (-0.38, 0.24) | 84 | -0.42 | (-0.72, -0.11) |
| >18.99 | 75 | -0.02 | (-0.35, 0.31) | 81 | -0.19 | (-0.52, 0.14) |

Abbreviations: per- and polyfluoroalkyl substances, PFAS; polybrominated diphenyl ethers, PBDE; CI, confidence interval.

Models adjusted for maternal age, maternal race/ethnicity, pre-pregnancy BMI, maternal education, smoking status, parity, and food insecurity.

Table S9. Linear regression coefficients and 95% confidence intervals for the associations between tertiles of PFAS (ng/mL) and PBDE (ng/g lipid) concentrations in maternal serum and gestational age stratified by infant sex (N=506).

|  |  | **Male (N=233)** | |  | **Female (N=273)** | |
| --- | --- | --- | --- | --- | --- | --- |
| **PFAS** | **N** | **β** | **95% CI** | **N** | **β** | **95% CI** |
| PFOA |  |  |  |  |  |  |
| <1.40 | 69 | Reference | | 90 | Reference | |
| 1.40-0.96 | 79 | -0.24 | (-0.91, 0.43) | 82 | -0.31 | (-0.95, 0.34) |
| >0.96 | 74 | -0.11 | (-0.92, 0.70) | 85 | -0.11 | (-0.87, 0.65) |
| PFOS |  |  |  |  |  |  |
| <1.40 | 77 | Reference | | 84 | Reference | |
| 1.40-2.56 | 71 | -0.60 | (-1.29, 0.09) | 90 | 0.15 | (-0.50, 0.80) |
| >2.56 | 74 | -0.49 | (-1.25, 0.26) | 83 | 0.36 | (-0.40, 1.12) |
| PFHxS |  |  |  |  |  |  |
| <0.24 | 67 | Reference | | 90 | Reference | |
| 0.24-0.49 | 85 | 0.18 | (-0.49, 0.85) | 82 | 0.33 | (-0.34, 1.00) |
| >0.49 | 50 | 0.06 | (-0.76, 0.88) | 85 | 0.06 | (-0.75, 0.86) |
| PFNA |  |  |  |  |  |  |
| <0.24 | 70 | Reference | | 90 | Reference | |
| 0.24-0.49 | 112 | -0.34 | (-0.98, 0.30) | 119 | -0.19 | (-0.80, 0.42) |
| >0.49 | 40 | -0.67 | (-1.56, 0.22) | 48 | 0.03 | (-0.79, 0.85) |
| Me-PFOSA-AcOH |  |  |  |  |  |  |
| <0.04 | 78 | Reference | | 84 | Reference | |
| 0.04-0.06 | 65 | -0.45 | (-1.14, 0.23) | 96 | 0.56 | (-0.02, 1.14) |
| >0.06 | 79 | -0.60 | (-1.25, 0.05) | 77 | 0.79 | (0.15, 1.43) |
| *Total PFAS* |  |  |  |  |  |  |
| <2.62 | 74 | Reference | | 86 | Reference | |
| 2.62-4.83 | 75 | -0.70 | (-1.41, 0.01) | 85 | -0.08 | (-0.73, 0.57) |
| >4.83 | 72 | -0.64 | (-1.42, 0.15) | 86 | 0.16 | (-0.58, 0.89) |
| **PBDE** |  |  |  |  |  |  |
| BDE-47 |  |  |  |  |  |  |
| <8.47 | 74 | Reference | | 91 | Reference | |
| 8.47-14.22 | 75 | -0.04 | (-0.68, 0.60) | 85 | -0.08 | (-0.67, 0.52) |
| >14.22 | 71 | -0.65 | (-1.33, 0.03) | 81 | -0.31 | (-0.97, 0.34) |
| BDE-99 |  |  |  |  |  |  |
| <2.88 | 68 | Reference | | 93 | Reference | |
| 2.88-4.71 | 82 | 0.05 | (-0.60, 0.69) | 81 | 0.10 | (-0.52, 0.71) |
| >4.71 | 72 | -0.24 | (-0.95, 0.48) | 83 | -0.24 | (-0.85, 0.38) |
| *Total PBDE* |  |  |  |  |  |  |
| <11.38 | 71 | Reference | | 92 | Reference | |
| 11.38-18.99 | 76 | 0.03 | (-0.62, 0.67) | 84 | -0.25 | (-0.85, 0.34) |
| >18.99 | 75 | -0.55 | (-1.23, 0.14) | 81 | -0.26 | (-0.89, 0.37) |

Abbreviations: per- and polyfluoroalkyl substances, PFAS; polybrominated diphenyl ethers, PBDE; CI, confidence interval.

Models adjusted for maternal age, maternal race/ethnicity, pre-pregnancy BMI, maternal education, smoking status, parity, and food insecurity.
